# Synthetic biology to revive microbial natural product discovery

**DOI:** 10.1002/mlf2.12071

**Published:** 2023-06-22

**Authors:** Xiaoyu Tang

**Affiliations:** ^1^ Shenzhen Bay Laboratory Institute of Chemical Biology Shenzhen China

Microbial natural products (MNPs) and their derivatives have historically played critical roles in drug discovery. However, the discovery of novel MNPs over the last 60 years, through traditional strategies, has declined significantly. Advances in genome sequencing technologies have rapidly changed the direction of natural product research in recent years, providing opportunities to revive the natural product discovery pipeline. Here, I highlight several paradigms combining genomics with synthetic biology to enable MNP discovery and envision future opportunities.

Diffusible and microbe‐associated small molecules, also known as MNPs, often play important roles in mediating intra‐ and/or interspecies interactions in complex ecological systems. In turn, these interactions can help stabilize ecosystems and affect the health and disease of hosts. Thus, MNPs have been critical in human medicine, serving as an important source for the discovery and development of drugs to treat infectious diseases, cancers, high cholesterol, and so on^1^. In addition, such compounds have long been used in developing agrochemicals and food additives^2^, ^3^. However, despite the enormous contributions of MNPs to our society, how these chemicals are discovered from nature has not significantly changed over the past century. Most practitioners, whether in academia or industry, adopt a “grind and find” approach involving chemical and/or biological screens to discover MNPs from field‐collected resources or laboratory‐cultured organisms, because of which the discovery rate of new natural products has dramatically fallen in the past 30 years. As a consequence, the high rediscovery rate of known MNPs was a major reason why pharmaceutical companies either terminated or dramatically reduced their natural product discovery programs.

In general, natural products are genetically encoded and biosynthesized endogenously. Interestingly, genes responsible for encoding biosynthetic enzymes are typically arranged in clusters on the genome of microbial producers (bacteria and fungi), referred to as biosynthetic gene clusters (BGCs). To produce natural products, biosynthetic enzymes work sequentially to form an assembly line, where pools of simple chemical building blocks, such as amino acids, fatty acids, and sugars, are activated and incorporated to form the final molecules. Over the past few decades, the natural product biosynthesis community has dedicated tremendous efforts to discovering and elucidating natural product biosynthetic enzymes, which has advanced our biosynthetic knowledge to the point where we can predict almost any type of BGC (or even their putative chemical structures) from sequence data. In addition, various genomic and metagenomic data are being rapidly accumulated in public databases through advanced sequencing technologies, with close to 550,000 complete and draft sequences of microbial genomes in the NCBI database as of April 2023. Recent bioinformatics efforts have revealed that only a small fraction of the natural product biosynthetic potential of most organisms has been realized, suggesting that current discovery methods have significantly underrepresented the chemical breadth of bacteria, fungi, and other higher organisms^4^. For instance, a current study estimates that only 3% of the natural products potentially encoded in bacterial genomes have been experimentally characterized^5^. To reinvigorate the MNP discovery pipeline, new discovery methods involving orthogonal approaches are urgently needed.

As MNPs are all genetically encoded organic molecules, synthetic biology can be used to convert biosynthetic potential into chemical realities. Synthetic biology involves reprogramming pathways or organisms for desired purposes by altering genetic codes. To apply synthetic biology to natural product discovery, the ability to manipulate genes involved in natural product biosynthesis, either homologously or heterologously, is a prerequisite. Many previous attempts to engineer biosynthetic pathways in nonmodel organisms have been either defeated or ended up with time‐consuming procedures. In recent years, the discovery of the clustered regularly interspaced short palindromic repeats (CRISPR)/Cas (CRISPR‐associated proteins) gene‐editing systems has revolutionized the field of natural product discovery. As an example, the Huimin Zhao group leveraged the CRISPR‐Cas9‐mediated knock‐in strategy to activate silent BGCs in *Streptomyces* species^6^. By replacing the promoter regions of the main biosynthetic operons of silent BGCs with constitutive promoters, they observed the production of various natural products from these BGCs, including type I, II, and III polyketide synthases (PKSs), nonribosomal peptide synthetase (NRPS), hybrid PKS–NRPS, and phosphonate clusters. Another example involves the use of the CRISPR‐Cas9‐mediated knockout strategy to terminate the production of the most frequently rediscovered antibiotics in actinomycete strains, resulting in the rapid discovery of some rare and previously unknown variants of antibiotics^7^. These two strategies represent a new model for discovering new natural products using the concept of synthetic biology.

**Figure 1:**
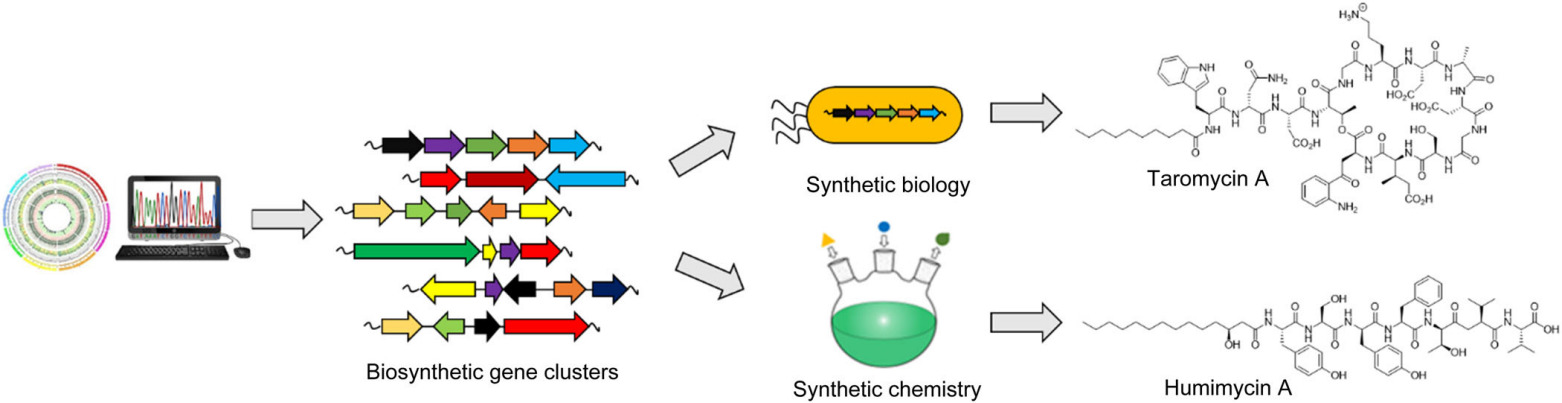
New paradigms for discovering microbial natural products.

In addition to manipulating BGCs in wild‐type hosts, another approach involves the expression of foreign biosynthetic pathways in a well‐established and genetically amenable host. However, this approach demands the cloning of the entire BGC into suitable expression vectors. Recently, several new strategies, such as full‐length RecE‐mediated linear‐plus‐linear homologous recombination, transformation‐associated recombination (TAR) cloning, Gibson assembly, and their combinations, have been developed to accelerate the BGC cloning process^8^. As an example, Moore and colleagues used TAR cloning in yeast to directly clone an orphan BGC from a marine actinomycete strain. After refactoring the regulatory genes in yeast, the BGC was successfully expressed in the model expression host *Streptomyces coelicolor*. This resulted in the identification of the dichlorinated lipopeptide antibiotic taromycin A (Figure 1), which is structurally similar to the clinically approved antibiotic daptomycin^9^. In another case, they heterologously expressed and engineered a streptophenazine pathway in *S. coelicolor* via TAR cloning, resulting in the production of over a hundred chemicals^10^. This affirms that heterologous expression represents another approach to discovering, characterizing, and re‐engineering new pathways to developing new natural products using synthetic biology.

Currently, advances in synthetic biology methodology have arguably progressed to the point where synthetic biologists can program genetic codes from any type of BGC, regardless of cost and time. However, converting genetic materials into chemical entities remains challenging, roughly requiring four steps (transcript, translation, biosynthesis, and detection) without a bug in the program. “Uncertainty is there, but can we escape from the need for gene expression to get the chemical entities?” This was a question posed by Sean Brady and colleagues at Rockefeller in 2016 when they proposed a synthetic–bioinformatic strategy for discovering new antibiotics^11^. This approach requires the bioinformatical prediction of chemical structures from a primary DNA sequence based on the existing biosynthetic knowledge, followed by chemical synthesis of the predicted structures. Through this approach, the scientists synthesized a group of new peptide antibiotics (named humimycins; Figure 1) on the basis of the biosynthetic information from the human microbiome^11^. Although the authors could not find the authentic products from the associated BGC, humimycins showed promising activities in a mice model to treat methicillin‐resistant *Staphylococcus aureus* infection, presumably targeting the biosynthesis of bacterial peptidoglycan by inhibiting lipid II flippase. Following a successful proof of concept, the Brady group used the synthetic–bioinformatic approach to identify three new types of peptide antibiotics, including a potent dual topoisomerase I/II inhibitor^12^, a group of menaquinone‐binding antibiotics^13^, and a series of new colistin congener against Gram‐negative pathogens^14^. In another recent study, Wang, Chen, and colleagues developed a machine‐learning approach for mining antimicrobial peptides (AMPs) from the human gut microbiome. Based on the amino‐acid coding information, they chemically synthesized 216 AMPs and developed 181 AMPs with antimicrobial activity^15^.

In the past, synthetic chemistry was frequently used for the total synthesis and diversification of characterized natural products after the corresponding chemicals were identified. Today's synthetic chemists can decode the templates of nature's chemical repositories based on genomics, bioinformatics, and chemistry, not to mention further expansion of the topological and functional properties of chemical entities. Although this approach is currently limited to BGCs with well‐established biosynthetic logic (such as NRPSs or ribosomally synthesized peptides), we envision that biosynthetic knowledge accumulation, along with the development of bioinformatic prediction algorithms, will extend the chemical ability to access more potential drug leads from nature.

Promising life‐saving medicines exist; we need to integrate them from various perspectives to maximize our chances of obtaining them. The combination of synthetic biology and synthetic chemistry may usher in a new era in natural product discovery.
